# 

**Published:** 1891-10-24

**Authors:** 


**Notices of Births, Deaths, and Marriages,** are inserted in this column at a charge of 2s. 6d. for an announcement not exceeding four lines.

### NOTICE TO CORRESPONDENTS.

All MS., Letters, Books for Review, and other matters intended for the Editor should be addressed The Editor, The Lodge, Porchester Square, London, W.

The Editor cannot undertake to return rejected M.S., even when accompanied by stamped directed envelope.

**Notice to Advertisers and Subscribers.**

All Advertisements, Orders for Copies of The Hospital, and Business Communications should be addressed to The Publisher, *not to the Editor*, The Hospital, 140, Strand, W.C.

The Cost of Subscription, post free, is as follows:—Per week, 1½d.; per quarter, 1s. 8d.; per half-year, 3s. 3d.; per annum, 6s. 6d. Foreign:—Per annum, 8s. 8d.; payable, in all cases, in advance.

**"Burdett's Hospital Annual," 1891—1892.**

Third year of publication. 600 pp. Boards, gilt lettering. A most complete guide to British and Colonial Hospitals, Dispensaries, Nursing and Convalescent Institutions, and Asylums. Price 3s. 6d. Published by The Hospital (Limited), 140, Strand, W.C.

**NOTICE.—The Index for Vol. X. (ending September, 1891), is on sale at the Office of this Journal, price 2½d., post free.**

### General Charities.

[This Column is devoted to an account of work of charitable institutions other than Medical Charities. The Editor will be glad to receive reports or news of work at 140, Strand. *Philanthropy* should be plainly written on the envelope.]

The following, among other institutions, have been benefited under the will of the late Mrs. Elliott, of Bath: the Metropolitan Association for Improving the Dwellings of the Poor, the London Congregational Ministers' Superannuation Fund, £1,000 each; the Haverstock Hill Orphan Home, the Shipwrecked Mariners' Society, the British and Foreign Bible Society, and the Protestant Reformation Society, £500 each.

Philanthropic effort is now entering on a period of renewed activity with the approach of winter. The *Yorkshire Post* has set the ball rolling on behalf of the National Lifeboat Institution, and has procured a considerable sum from its readers towards the extinction of the large deficit to which we alluded last week. Now this good example has been set, it is to be hoped that the public, who cannot plead ignorance of the state of affairs, will come forward handsomely.

An infinite number of institutions throughout the country owe a debt of gratitude to the late Miss Hannah Pickard, of Green Mount, Ossett, Yorkshire. She has bequeathed the sum of £1,000 to the Royal National Lifeboat Institution, in aid of the maintenance of the five boats for which the late Mr. Andrew Pickard left £5,000 last year; and amongst other societies in London which have received legacies are the Society for Providing Drinking Troughs for Cattle in Towns, £1,000; the London Home of Rest for Horses, the British and Foreign Bible Society, £500 each, and the London Mission to Outcast Poor, £250.

Like many other Institutions, the **Universal Beneficent Society** has suffered recently from a considerable decrease in its funds. The extent of this decrease is such as to have prompted the Council to make an earnest appeal for help to their generous subscribers, and to the public generally, in order that they may be able to deal satisfactorily with some at least of the urgent cases which come before them. Many distressing cases are now on the books owing to the serious outbreak of influenza, which has attacked the poor as well as the rich. It should be remembered that the poorer classes of society have suffered deeply from the ravages of this disease, partly from not being able to keep away from work sufficiently long to enable them to regain their strength. The result has too often been the death of the bread-winner of the family, who has left behind him a widow and children totally unprovided for. Unfortunately, the influenza is still with us, and is not unlikely to break out again with renewed vigour during the winter. The main object of the Society is to afford relief to persons who are not classed as the poor in the general acceptation of the term, but who, nevertheless, suffer privations all the more severe because of the position which they formerly held. In other words, people who are found to be in a state bordering on destitution which, if they are not adequately relieved, as the Society attempts to do by way of annuities of £15, by temporary assistance, or by procuring the admission of orphans and destitute children into suitable homes, leads them to absolute pauperism. As the Society has to depend solely upon voluntary contributions to enable it to carry out the work for which it was founded, the Council will be grateful for donations, which should be sent to the Secretary, Mr. G. Stormont Murphy, 15, Soho Square, W.

The title of **The London Female Preventative and Reformatory Institution** practically explains its objects, which are to help friendless and fallen young women. There are several such institutions, meritorious in their origin, but not always happy or successful in their administration. How best to go about reforming women who have become the scapegoats of society has always been a vexed question, and one which has brought despair to the hearts of many who strive after the welfare of their less fortunate fellow beings. The advocates of bringing religious means to bear have generally had to confess failure in their efforts, whereas those who have endeavoured to base their work on the principle of making the surroundings of these women cheerful and happy can boast of the greatest success. The London Female Preventative Institution gives preference in its operations to country girls and orphans, and, where able, restores to their homes young girls who are left adrift in London. According to the report presented to the meeting at the Mansion House last week on behalf of the Society, the number of applications for help is largely increasing, a fact which necessitates an appeal for further support from the public. Since the establishment of the society in 1857, 13,487 cases have been helped. At the present time there are seven homes and one "open all night refuge," providing altogether for 240 inmates; in 1857 there was one home for 16 inmates only. It is a popular belief, and we are not sure that it is not a fallacy, that the country girl is not as capable of looking after herself as her urban sister; but the average country girl of the present day, though not to all appearances as sharp as the town girl, is generally endued with quite as much common sense. Anyhow, the view put forward by the Lord Mayor, that going on the streets is the only end for young girls from the country who are left to their own resources in London, is perhaps rather an extreme one. That there is ample scope for the operations of the Society is, however, only too evident.

### Scraps and Gleanings.

Scarlet fever has broken out at Aberdeen.

Mr. Lees has presented Oldham Infirmary with the munificent gift of £10,000.

Birmingham Musical Festival will be able to award £5,500 profits to the local charities.

The Convalescent Home for Women, at Southsea, is now established in its new quarters.

Lady Malmesbury has laid the foundation-stone of the new church of St. Andrew, Bournemouth.

St. Leonards has now a further attraction for invalids in the form of the new pier opened by Lady Brassey last month.

Waterlow Park, Highgate, was opened to the public on Saturday last by Sir John Lubbock, on behalf of the County Council.

A serious outbreak of diphtheria has occurred at Heywood, and the public schools are being closed in consequence. The deaths are numerous.

The Cork Fever Hospital is very proud of having "captured" a small-pox case. They now appeal confidently for funds, and for the consideration of the people of Cork.

Lady Henry Somerset will, it is stated, preach the annual sermon in Fremont Temple, Boston, in connection with the Women's Christian Temperance Union of America.

It has been pointed out that a great need exists at Crewe for additional hospital accommodation. The London and North-Western Railway own the only hospital existing there.

A lad named Kitchin has, it is stated, had an injured portion of his brain removed. The surgeons regarded the case as hopeless, but although he was paralysed he ultimately recovered.

English visitors frequenting the Riviera will be glad to hear that the report relative to the drainage of Cannes, presented to the Paris Consultative Committee of Hygiene, has been adopted.

Investigation on the subject of self-purification of rivers in Germany shows that the pollution of rivers by the large populations of towns disappears in the waterflow a few miles down the stream.

The Board of Superintendence of the Rotunda Lying-in Hospital, Dublin, have submitted their report to Parliament, as the present election of Governors and Master is felt to be objectionable.

Queen Christina has been to the Charity Hospital at Burgos to visit the Spaniards who were injured in the recent railway disaster. Later in the day Her Majesty visited Mr. Seymour Lucas and Mr. Fletcher.

The new nurses' home, additional operating theatres and wards, at the Edinburgh Royal Infirmary will cost not less than £15,000. The new nurses' home is nearly roofed in, and ought to be ready for occupation in a few months.

A patient in Guy's Hospital, named Guest, shot himself last week while in bed in one of the wards. The diseases from which he suffered were very painful. Great surprise is expressed that the possession of the pistol was not discovered.

Last year there were 1,170 persons prosecuted for the illegal practice of medicine in Bavaria, namely, 861 men and 309 women. "Secret remedies and sympathy" had 117 adherents; homœopathy, 71; magnetism, 12; and electro-homœopathy, 9.

It strikes us as strange that a patient is reported as discharged from the Devonshire Hospital, Buxton, because "infested with vermin." The system of supplying patients with post cards, to report on their condition after leaving the hospital, is a good one.

Steps are being taken to establish a private hospital in Cookstown for treatment of paying patients. The county infirmaries are situated at considerable distances—at Omagh and Londonderry—and patients generally go to Belfast. The proposed hospital, it is thought, would be self-supporting.

Dr. Lehner, a chemist, of Augsberg, Germany, has invented a process of making artificial silk, which is about to be manufactured on a large scale. The material is very like real silk, and is about two-thirds as strong, while its colour can be made superior to that of the natural fibre.

At a meeting of the Health Committee of the Birkenhead Town Council, the position of Dr. Vacher, the medical officer of health was fully considered in his relations to the Fever Hospital, and his resignation was accepted. The resignation has yet to come before the Council before ratification.

The Wycombe Cottage Hospital is now undergoing the addition necessary to meet the terms on which the balance of the Beaconsfield Memorial Fund was handed over to the trustees. Mr. Coningsby Disraeli, nephew and heir of the late Earl, has consented to perform the re-opening ceremony.

At St. Thomas's Hospital a fit subject for relief was allowed to go away untended after examination by a student, who declared that no injury existed. The patient in question, a child, was found by a private practitioner to have broken its thigh. It finally gained admittance into St. Thomas's, where it died.

Samuel Hurley is a specialist of distinction; he is an adept at producing what are known in mendicant circles as "soapy fits"; so much so that amongst mendicant circles he has gained the proud title of the "Soapy Fits King." His successful career has at last terminated, and he is now under charge for imposture and vagabondage.

It has been proposed to increase the staff of surgeons at the Bedford Infirmary. The Honorary Staff objects on several grounds, and points out that in the Bedford Infirmary with the present staff there are only 17 beds to each physician and surgeon, as against 29 and 25 respectively in the larger and smaller hospitals of many other places.

A convalescent home on a large scale is now being built at Parkwood, Kent, for the reception of 120 patients from the principal London hospitals, the majority of which have no convalescent home of their own. The building, which stands in fifty acres of ground, is the gift of Mr. Peter Reid, who gave £100,000, to which an anonymous donor added the sum of £50,000.

### The Student of Medicine.

[All communications for this Supplement should be addressed to The Editor of The Hospital, at 140, Strand, with the words, "Students' Column," written in the left hand top corner of the envelope.]

### APPOINTMENTS.

At the New Town Dispensary, Edinburgh, Alex. Miles, M.D. Edin., F.R.C.S., has been appointed medical officer. At the City of London Hospital for Diseases of the Chest, Charles H. McLlwaiwraith, M.A., M.B., C.M., Glasgow, has been appointed house physician. At the Edinburgh Royal Infirmary, David James Brackenridge, M.D., L.R.C.P., M.R.C.S., has been appointed senior ordinary physician; Alexander James, M.D., C.M., F.R.C.P. Edin., senior assistant physician; and Andrew Smart, M.D., F.R.C.P. Edin., junior ordinary physician. At the Macclesfield General Infirmary, A. J. Beasley, M.B., C.M. Vict., has been appointed junior house surgeon, and Oswald Rees, M.B., C.M., senior house surgeon. At the Newcastle-on-Tyne Ear and Throat Hospital, John Burdon, L.R.C.S., L.R.C.P.Edin., has been appointed surgeon. At the Western Ophthalmic Hospital, Marylebone Road, Kenneth E. Campbell, M.B., F.R.C.S. Eng., has been appointed surgeon, and Frank Haydon, L.R.C.P. Lond., assistant surgeon. At the Scarborough Hospital and Dispensary, W. S. Carpenter, M.R.C.S., L.R.C.P., has been appointed house surgeon. At the Middlesex Hospital, Arthur Francis Voelcker M.D., B.S. Lond., M.R.C.P., has been appointed pathologist and curator of the museum. At the Brennock County and Borough Infirmary, J. T. Grey, M.R.C.S., L.R.C.P., has been appointed house surgeon. At the Burton-on-Trent General Infirmary, R. Meredith Little, M.R.C.S., L.R.C.P., has been appointed house surgeon. At the Victoria Hospital for Children, Hull, C. H. Milburn, M.B., B.S. Durh., has been appointed surgeon. At the Intern Department of the Belfast Lying-in Hospital, H. D. Oshorne, L.R.C.P., L.R.C.S. Edin., has been appointed assistant physician. At the Islington Dispensary, Richard Arthur Smith, M.R.C.S., L.R.C.P., has been appointed resident medical officer. At the Bolton Infirmary and Dispensary, G. Wilkinson, B.A., M.B., C.M., has been appointed junior house surgeon. At the Ancoats Hospital, Manchester, T. S. Worboys, M.R.C.S., L.R.C.P., has been appointed physician.

### VACANCIES.

The following vacancies are announced this week, the amount of salary in each case being given after the name of the institution:

**Resident Medical Officers.**

Chester General Infirmary, Visiting Surgeon—Salary £80, with board, &c.
Eastern Counties Asylum for Idiots, Colchester, Resident Medical Attendant—Salary £100, and board, &c.
East London Hospital for Children, Shadwell, E., House Physician—Board, &c.
General Infirmary Leeds, Resident Obstetric Officer, House Physician, and two House Surgeons—Board, &c.
General Infirmary, Leeds, Resident Medical Officer at the Ida Hospital Honorarium for six months, £25.
Hospital for Diseases of the Throat, Golden Square, Resident Medical Officer—Salary £50, with board, &c.
Inverness District Asylum, Assistant Medical Officer—Salary £100, and board, &c.
Kent County Asylum, Chatham, Junior Assistant Medical Officer—Salary £125.
London Fever Hospital, Liverpool Road, N., Assistant Resident Medical Officer.
North-West London Hospital, Kentish Town Road, Resident Medical Officer—Salary £50, and board, &c.
North-West London Hospital, Kentish Town Road, Assistant Resident Medical Officer—Board, &c.
Royal Alexander Hospital for Sick Children, Brighton, House Surgeon—Salary £80, with board and washing, &c.

**Non-Resident Medical Officers.**

Charing Cross Hospital, Medical Registrar—Salary £40.
City of London Hospital for Diseases of the Chest, Victoria Park, Assistant Physician.
Guy's Hospital, Assistant Anæsthetist.
Hastings, St. Leonards, and East Sussex Hospital, Honorary Assistant Physician.

